# Can Mobile Technology Help Prevent the Burden of Dementia in Low- and Mid-Income Countries?

**DOI:** 10.3389/fpubh.2020.554938

**Published:** 2020-11-12

**Authors:** Bruno Bonnechère, Barbara J. Sahakian

**Affiliations:** ^1^Department of Psychiatry and Behavioural, Clinical Neuroscience Institute, University of Cambridge, Cambridge, United Kingdom; ^2^Public Health School, Université Libre de Bruxelles, Brussels, Belgium

**Keywords:** cognitive impairment, low income countries, cognitive training, new technologies, apps and smartphones

## Context

According to the World Health Organization (WHO), the global population aged above 60 years will double in size by 2050, with an estimated total of about 2 billion people. The increase in life expectancy, combined with unhealthy behaviors and physical inactivity (1), is linked to an increase in non-communicable diseases (NCDs). Additionally, related to aging but not yet considered to be an NCD is cognitive decline, which is an important medical condition resulting in a significant loss of autonomy.

Globally, the current number of people living with dementia is estimated to be 50 million, with nearly 10 million new cases every year, and representing a serious public health problem (2). Identified as one of the major challenges of the twenty-first century, the WHO proclaimed that cognitive decline and dementia are a mental health global priority.

The burden of cognitive diseases for patients, relatives, and nations is thus a major public health problem that must be addressed (3–5). In addition, there is a significant impact on well-being for both patients and their relatives (6, 7). These concerns in developed countries regarding the increased incidence of NCDs are now also of significant importance for mid- and low-income countries (8).

In this paper we aim to summarize the current level of evidence supporting the use of mobile technology to prevent dementia in low- and mid-income countries by undertaken a narrative review. Obstacles and future directions are also discussed.

## A Lack of Resources

Medical resources include professional health workers and access to infrastructure, devices, and drugs; however, professionally trained health workers are unequally distributed across the world. Countries with the lowest relative need have the highest numbers of health workers, while those with the greatest burden of disease must endure with a much smaller health workforce. The African continent suffers more than 24% of the total global burden of disease but has access to only 3% of trained health workers and <1% of the world's financial resources (9). Although the majority of these insufficient human and material resources are allocated to infectious diseases, only a small fraction is invested in mental health, including cognitive disorders.

Traditionally, a diagnosis of dementia has been primarily based on clinical criteria and anamnesis. At present, the diagnosis is made with imaging techniques, as reflected in the updated diagnostic criteria (10). A major barrier to patients' assessments and rehabilitation, as stated by the WHO, is the lack of access to available clinical centers and neuroimaging techniques, particularly for those living in low- to mid-income countries or rural areas. This causes the gap to widen further (11).

**Figure 1:**
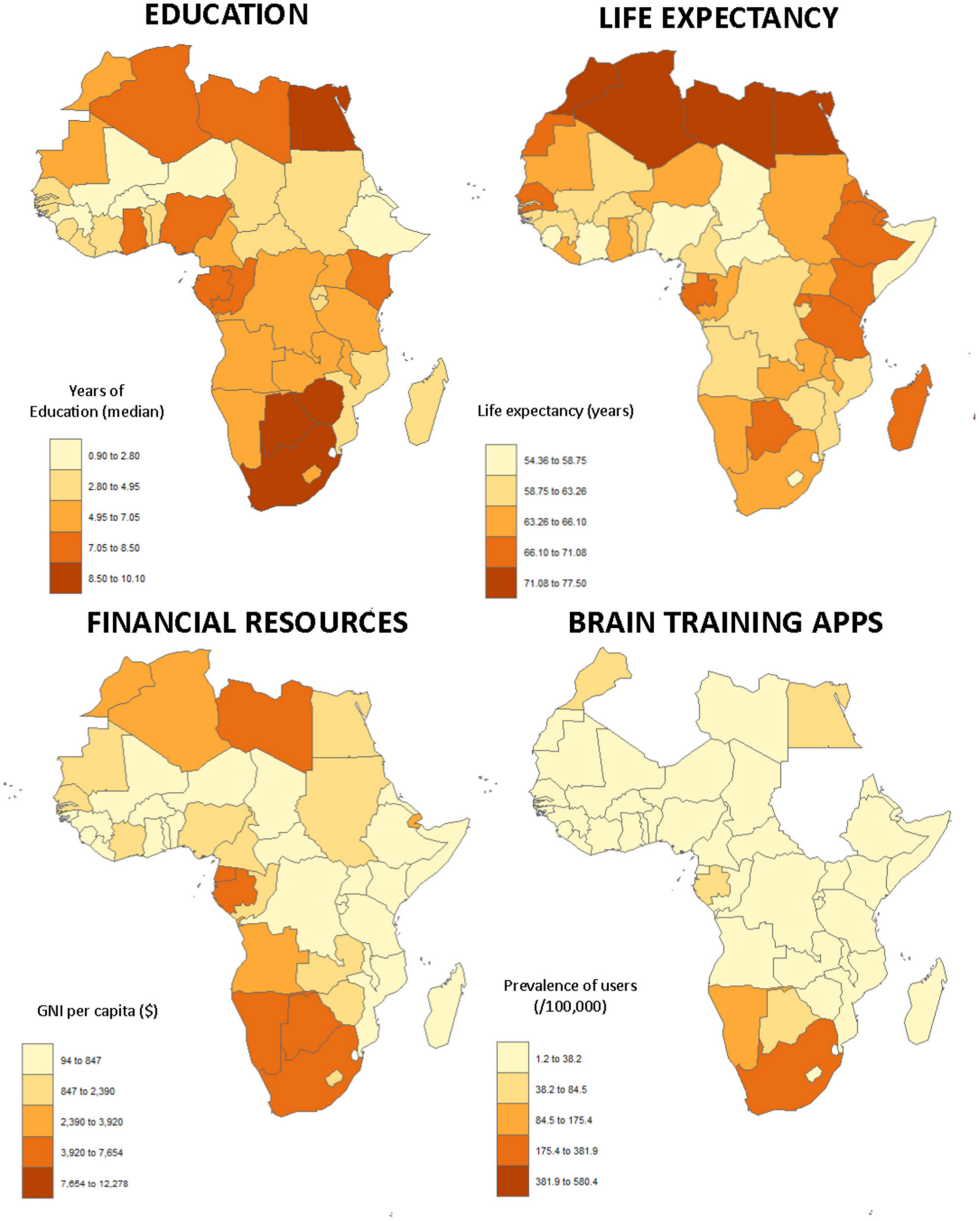
The top figures present two of the most important risk factors of dementia aggregated at the country level: low educational level (left) and life expectancy (right). The figures at the bottom relate to the use of technology, with GNI per capita on the left and the prevalence of users of the application per country on the right. These original figures were constructed in R using the “cartogram” package. Data on the median age of the populations and the number of inhabitants were obtained from the World Factbook (16), GNI from the World Bank (The World Bank Group^2^), the level of education from the Demographic and Health Survey Program (The DHS Program^3^), and the life expectancy from Worldometers^4^.

Another important issue is that the education system is frequently less accessible in low- to mid-income countries (Figure 1). Education and life-long learning are well-known and important modifiable risk factors (12, 13) and enhance cognitive reserves, thus appearing to provide some resilience against dementia (6, 14).

A solution that could partially reduce this health inequality could be provided by advances in technology because mobile phone penetration is high, even in low- to mid-income countries, thus ensuring access to diagnostic and treatment apps for a variety of physical and mental health problems (15).

## Current Situation

The risk of dementia increases with age and the majority of cases, at least in developed countries, do not appear until age 70 (5). With a mean life expectancy of 64.9 (5.5) years, but an overall median age of 20.5 (4.4) years, Africa has the youngest population of the world's continents (16). Due to its low average age, the frequency of dementia is still low in Africa. However, as presented in Figure 1, important disparities exist between the countries. In a meta-analysis summarizing the results of 11 cross-sectional studies (Benin, Botswana, the Central African Republic, the Congo, and Nigeria), which enrolled a total of approximately 10,500 participants, the authors found the prevalence of dementia ranged from 0 to 10.1% within the included studies. The prevalence of cognitive impairment ranged from 6.3 to 25% (17). It is important to note that these numbers are difficult to compare with those of European countries or the United States because the age of the populations studied is different (i.e., in the meta-analysis, the authors described elderly adults as those older than 50 years). However, it is expected that by 2050 about two-thirds of dementia will occur in low- or mid-income countries, representing, therefore, a huge public health challenge (18).

## Potential Solution

Mobile technology has spread rapidly around the globe. According to an estimation made by the Pew Research Center, more than 5 billion people own mobile devices (half of these are smartphones). The evolution of the use of mobile technology differs by country: a median of 76% of the population in advanced economies have smartphones, compared with a median of only 45% in emerging economies. Interestingly, smartphone ownership also continues to grow in emerging nations (19).

The impact of digital health on patient care is rapidly accelerating with the increased adoption of mobile health apps and wearable sensors. In 2017, the number of health-related mobile applications available to consumers surpassed 318,000—nearly double the number available only two years earlier (20).

Digital health solutions include apps that can inform patients about medical conditions, try to modify their health-related behavior, track, and monitor conditions, or assist in training (21). This is a low-cost solution that does not require immediate access to healthcare clinics or workers because it is self-driven and readily available.

For cognition, most of the apps developed are used for cognitive training; see Cochrane reviews (22–24). One of the key difficulties reported in the reviews is that many of the studies had large numbers of dropouts (up to 40%). Despite this, a recent meta-analysis concluded that commercially available computerized cognitive games are effective in improving cognitive function in participants without cognitive impairment aged over 60 years (25) Training using a game app can motivate healthy people (26) and especially those with neurodegenerative diseases (27, 28) or psychiatric disorders, in which apathy or negative symptoms may be present (29, 30).

Notably, all of these studies were carried out in high-income countries where, due to different levels of cognitive simulations or educational backgrounds, findings could be different if the studies were conducted in low- to mid-income countries.

A salient aspect of game app training is that it can be combined with cognitive assessment to provide screening and follow-up of the cognitive function on a regular basis. This can be done at a lower cost and without the need of a healthcare professional because the results of the games are correlated with clinical scales (31) and the age of the participants (32).

## Current Use

To evaluate the global use of brain training mobile apps, we analyzed the number of users from the leading company in the field per continent (Peak Brain Training^1^). In Africa, the median prevalence of people using the app is 10.98 per 100,000 inhabitants (p25 = 4.27; p75 = 50.48/100,000); 110.95 (36.30; 358.01) in Asia; 744.87 (197.58; 1776.97) in Oceania; 869.11 (414.45; 1991.49) in America; and 2096.94 (690.55; 4081.38) in Europe.

To further assess the use in Africa, we collected data (aggregated at the country level) on the level of education using data from the Demographic and Health Survey Program (The DHS Program) and Gross National Income (GNI) from the World Bank (The World Bank Group); results are presented in Figure 1. We did not find a direct significant association between the median age of education and the use of the apps (β = 3.65 [95% CI: −4.35–11.67], *p* = 0.35), but a strong relationship between GNI and the use of app (β = 0.028 [95% CI: 0.016–0.039], *p* < 0.001).

## Problems to Solve

There are two main issues related to the use of technology.

The first is material; in addition to hardware (acquisition and maintenance) and internet issues, a significant factor currently associated with the use of apps in Africa is GNI. Although the software can be easily distributed for free, doing so is more problematic for the hardware. However, the non-profit organization “One Billion” aims to provide tablets with specific programs for teaching one billion children to calculate and read in their own language (onebillion^5^ Amongst the identified modifiable risk factors of dementia, the level of education is one of the most important (13), and the mean level of education remains low in Africa (Figure 1). Education enhances the cognitive reserve and appears to provide protection from dementia (14). This project could not only improve the education and cognitive levels, but should also allow greater use of the technology.

The second limitation concerns the acceptance of the technology. Targeting the elderly cohort in low-income countries represents a double challenge: cultural and generational.

The acceptance of new technologies, as part of medical diagnosis or intervention, is a major problem because a large part of the population continues to rely on traditional medicine (33). Another important potential limitation is that relatives often hide cognitive problems due to stigma (34); therefore the prevalence of dementia is probably underestimated and many patients do not receive sufficient medical attention. Beliefs, cultural aspects, and traditions should be integrated into the proposed solutions to increase the adherence to diagnosis, treatment, and follow-up (35). Furthermore, the solution developed should be, as far as possible, not influenced by the educational status of the subjects because it is known that education has an important effect on cognitive assessment (36). There are also culture effects (37) and language effects (38). The games are short, culture-free, and unbiased instruments for improving cognitive functions and can be scaled in difficulty to ensure motivation stays high regardless of education level. For the training component, integrating the cultural aspect into the games increases users' involvement and participation (39).

On a global scale, attempting to address the issue of aging with technology is also challenging. Four key categories of aging barriers influencing the usability of digital health were identified: cognition, motivation, physical ability, and perception (40).

Despite the potential positive effect on aging, the main users of digital health apps are, currently, individuals who are younger, have more education, reported excellent health, and have a higher income (41).

Another issue, not specific to the use of new technologies or cognitive impairment, is the difficulty of translation from research projects to daily use of the developed solutions. A key challenge is moving digital health approaches from pilot projects to scalable national programs (42).

## Conclusion and Call to Action

Ooms et al. makes the important ethical point that If health is a human right and if human rights are “rights held by individuals simply because they are part of the human species,” then all people, wherever they live, should be entitled to the same collective efforts that can protect or improve their health (43). The WHO's comprehensive mental health action plan 2013–2020 includes the need to implement strategies for promotion and prevention in mental health and to strengthen information systems, evidence, and research for mental health (44).

Enormous disparities exist between high- and low- or mid-income countries in terms of health and, in particular, mental health and neurodegenerative diseases (3). Medical apps and digital health could help resolve many of the problems faced by users from low- to mid-income countries by informing, training, and assessing their cognition for a relatively low cost on a large scale. There is a growing number of evidence-based cognitive training game apps for healthy young people (26), people with schizophrenia (30), and older people with amnestic mild cognitive impairment (28). New technologies need to be harnessed to improve global health care and to reduce the impact of cognitive impairment in chronic neuropsychiatric disorders, including amnestic mild cognitive impairment and mild Alzheimer's disease, schizophrenia, and attention deficit hyperactivity disorder.

Although technological devices cannot solve all of the health problems of low- and mid-income countries, in the absence of effective low-cost and accessible treatments for cognitive and motivational deficits, these mental health apps could be greatly beneficial. To develop innovative, effective solutions adapted for neurological and psychiatric patients whose cognition, quality of life, functionality, and well-being are impaired, researchers, clinicians, and app developers will need to collaborate.

Technological solutions are already available for cardiovascular diseases (45) and cancer (46), but are not being used widely for cognition or mental health, particularly not in Africa, as presented in this paper. It is time that mental health is considered equally as important as physical health. Utilizing innovative technologies could greatly improve cognition and well-being globally (6).

## Author Contributions

BB and BJS contributed equally to the writing of this manuscript. All authors contributed to the article and approved the submitted version.

## Conflict of Interest

BJS acknowledges previous technology transfer, via Cambridge Enterprise, to Peak for the Wizard and Decoder apps. The remaining author declares that the research was conducted in the absence of any commercial or financial relationships that could be construed as a potential conflict of interest.

## Footnotes

^1^https://peak.net/

^2^https://data.worldbank.org/indicator/NY.GNP.MKTP.CD

^3^https://dhsprogram.com/

^4^Worldometers Life Expectancy of the World Population. Available at: https://www.worldometers.info/demographics/life-expectancy/ (accessed September 2, 2020).

^5^https://onebillion.org/impact/partners-and-projects/
